# Nrt1 and Tna1-Independent Export of NAD^+^ Precursor Vitamins Promotes NAD^+^ Homeostasis and Allows Engineering of Vitamin Production

**DOI:** 10.1371/journal.pone.0019710

**Published:** 2011-05-11

**Authors:** Peter Belenky, Rebecca Stebbins, Katrina L. Bogan, Charles R. Evans, Charles Brenner

**Affiliations:** 1 Departments of Genetics and Biochemistry and Norris Cotton Cancer Center, Dartmouth Medical School, Lebanon, New Hampshire, United States of America; 2 Departments of Biochemistry and Internal Medicine, Carver College of Medicine, University of Iowa, Iowa City, Iowa, United States of America; 3 Molecular Phenotyping Core, University of Michigan Nutrition and Obesity Research Center, Ann Arbor, Michigan, United States of America; Laurentian University, Canada

## Abstract

NAD^+^ is both a co-enzyme for hydride transfer enzymes and a substrate of sirtuins and other NAD^+^ consuming enzymes. NAD^+^ biosynthesis is required for two different regimens that extend lifespan in yeast. NAD^+^ is synthesized from tryptophan and the three vitamin precursors of NAD^+^: nicotinic acid, nicotinamide and nicotinamide riboside. Supplementation of yeast cells with NAD^+^ precursors increases intracellular NAD^+^ levels and extends replicative lifespan. Here we show that both nicotinamide riboside and nicotinic acid are not only vitamins but are also exported metabolites. We found that the deletion of the nicotinamide riboside transporter, Nrt1, leads to increased export of nicotinamide riboside. This discovery was exploited to engineer a strain to produce high levels of extracellular nicotinamide riboside, which was recovered in purified form. We further demonstrate that extracellular nicotinamide is readily converted to extracellular nicotinic acid in a manner that requires intracellular nicotinamidase activity. Like nicotinamide riboside, export of nicotinic acid is elevated by the deletion of the nicotinic acid transporter, Tna1. The data indicate that NAD^+^ metabolism has a critical extracellular element in the yeast system and suggest that cells regulate intracellular NAD^+^ metabolism by balancing import and export of NAD^+^ precursor vitamins.

## Introduction

**Figure 1:**
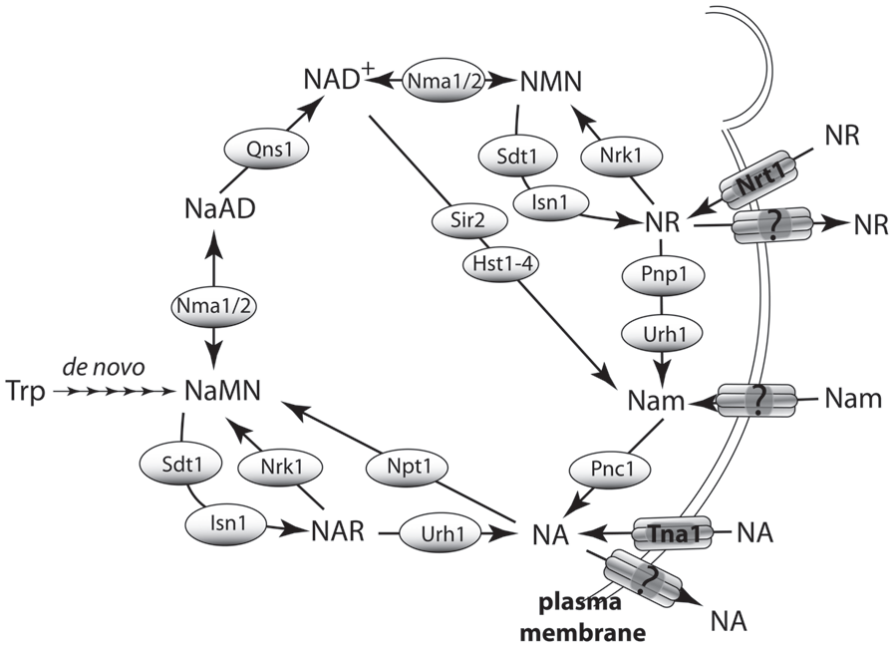
*S. cerevisiae* NAD^+^ Biosynthetic Pathways. The *de novo* biosynthetic pathway and salvage biosynthesis from nicotinic acid (NA) and nicotinamide (Nam) converge at NaMN. *De novo* biosynthesis consists of six enzyme-mediated transformation of tryptophan to NaMN. Salvage biosynthesis consists of a set of reactions from imported or salvaged NA, Nam or nicotinamide riboside (NR). NR is an unique precursor that can be converted to NAD^+^ without Qns1, the glutamine-dependent NAD^+^ synthetase.

Nicotinic acid (NA), nicotinamide (Nam) and nicotinamide riboside (NR) constitute the three salvageable NAD^+^ precursor vitamins in yeast. NA is imported by the high affinity major facilitator superfamily (MFS) transporter Tna1 (Figure 1) [1], [2]. However, at concentrations above 1 µM NA, Tna1-independent import is detectable [1]. NA is converted to NAD^+^ via the 3-step Preiss-Handler pathway (Figure 1) [3], [4]. Nam is converted to NA by nicotinamidase, Pnc1 [5], [6], for entry into Preiss-Handler salvage. There is no known Nam transporter.

NR is imported by the high affinity MFS transporter, Nrt1 [7] and converted into NAD^+^ *via* two distinct pathways. The first pathway utilizes the NR kinase, Nrk1, to produce nicotinamide mononucleotide (NMN), which is then converted into NAD^+^ [8]. The second pathway uses enzymes termed uridine hydrolase 1, Urh1, and purine nucleoside phosphorylase, Pnp1 [9], [10], to cleave NR into Nam plus a ribosyl product for Nam salvage. Urh1 has a strong preference for hydrolysis of NR over uridine, such that the term Urh1 is a misnomer [10].

In yeast, NR supplementation increases yeast NAD^+^ levels, promotes Sir2-dependent gene silencing and extends replicative lifespan [9]. Additionally replicative lifespan extension by calorie restriction requires intact NR salvage [11]. In vertebrate systems, NR has been shown to increase cellular NAD^+^ [12] and to protect against neuronal axonopathy [13]. Because of the distinct expression patterns of NR salvage enzymes and the lack of flushing, there is the potential for NR to emerge as a vitamin supplement and/or therapeutic agent with advantages over the commonly used niacins, NA and Nam [14] if problems in production can be overcome.

Nicotinic acid riboside (NAR) is an additional substrate of the Nrk enzymes [15] and Urh1 [10] that can be utilized as an NAD^+^ precursor by yeast (Figure 1) [10], [15]. However, efficient NAR utilization requires an ester modification [10], suggesting that NAR is an intracellular metabolite but not a vitamin that is transported into cells [11]. Recently, we and others described conditions in which yeast cells export NR [11], [16] and we developed a liquid chromatography-mass spectrometry (LC-MS) assay of the yeast NAD^+^ metabolome [17], which showed that Isn1 and Sdt1 function as NMN and nicotinic acid mononucleotide (NaMN) 5′-nucleotideases that are responsible for production of the NR and NAR metabolites [16].

The mechanism and purpose of NR export are unknown. Here we demonstrate and quantify that NR export is independent of Nrt1, the high affinity NR transporter. We also show that an NR non-salvaging and non-importing strain can be used to produce NR as an inexpensive, extracellular product. We establish that, like NR, NA is also an exported metabolite, and that production and export of extracellular NA from extracellular Nam depends on the intracellular nicotinamidase, Pnc1, but not the NA transporter, Tna1. The data indicate that NAD^+^ metabolism has an extracellular component that works in conjunction with intracellular metabolic pathways to regulate intracellular NAD^+^ precursor levels, store vitamins extracellularly, and potentially cross-feed other cells.

## Results

### NR export is Nrt1 independent

In yeast, NR supplementation can bypass the lethality of deletion of glutamine-dependent NAD^+^ synthetase, *QNS1*, [8] and extend replicative lifespan [9]. In addition to being an imported vitamin, NR is also an intracellular [17] and a secreted metabolite [11], [16]. On the basis of our discovery of the specific NR transporter, Nrt1 [7], we became interested in whether the transporter is responsible for the observed NR export activity. The NR-non-salvaging genotype *nrk1 urh1 pnp1* (strain PAB038, Table 1) has reduced intracellular NAD^+^ levels [9] and exports detectable levels of NR [11], [16]. To test whether Nrt1 is required for NR export from PAB038, we created strain PAB076 in which the *NRT1* gene in the PAB038 strain was deleted and replaced by a *URA3* marker gene.

**Table 1:** *S. cerevisiae* strains used in this study.

Strain	Genotype	Reference
BY4742	*MAT*α *his3Δ1 leu2Δ0 lys2Δ0 ura3Δ*	[24]
BY165-1D	*qns1::URA3* pB175	[8]
PAB008	BY4742 *tna1Δ::kanMX4*	This work
PAB011	BY4742 *nrt1Δ::kanMX4*	[7]
PAB038	BY4742 *pnp1Δ::kanMX4 urh1::NAT nrk1Δ::HIS3*	[9]
PAB041	BY4742 *pnc1Δ::kanMX4*	This work
PAB075	BY4742 *nrt1Δ::kanMX4 fun26Δ::URA3*	[7]
PAB076	BY4742 *pnp1Δ::kanMX4 urh1::NAT nrk1Δ::HIS3 nrt1Δ::URA3*	This work
PAB077	BY4742 *tna1Δ::kanMX4 pnc1Δ::URA3*	This work

**Figure 2:**
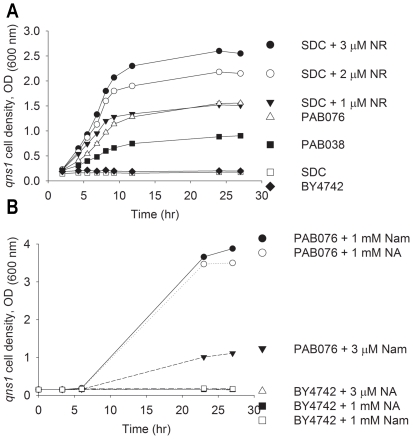
NR Export is Nrt1-Independent and Increased by NA and Nam Supplementation. A) NR exported by NR-accumulating strain PAB038 is not diminished but is rather increased by deletion of the NR transporter gene, *NRT1*, in strain PAB076. Conditioned media, collected from BY4742, PAB038 and PAB076 cells, grown in SDC media to a OD_600 nm_ of 3, were mixed 1∶1 with fresh media and evaluated for their support of *qns1* growth. The extent of *qns1* growth on these conditioned media samples was compared to *qns1* growth on fresh SDC supplemented with chemically synthesized NR (n = 3). B) NR export can be increased by supplementation of PAB076 with NA or Nam. Conditioned media, collected from BY4742 and PAB076 grown in SDC media supplemented with the indicated concentrations of NA or Nam, were evaluated for their support of *qns1* growth.

Extracellular NR can be detected using a *qns1* bioassay [11], [16] that relies on the NR auxotrophy of the *qns1* strain [8]. Qns1 activity is required for both *de novo* NAD^+^ biosynthesis and the utilization of NA and Nam (Figure 1). Because all other routes to NAD^+^ biosynthesis depend on Qns1, growth of the *qns1* strain can be used to detect the presence of NR [8]. In the *qns1* bioassay assay, strains tested for NR export are grown overnight in SDC. The growth of the *qns1* strain is then assayed in a 1∶1 mixture of conditioned media and fresh 2x SDC such that the extent of *qns1* growth is proportional to the extracellular concentration of NR. An estimation of extracellular NR can be made by comparing the growth of *qns1* in conditioned media to the growth of *qns1* in SDC supplemented with known amounts of pure NR. Based on the *qns1* bioassay, the deletion of *nrt1* from PAB038 does not reduce extracellular NR accumulation. On the contrary, NR levels are significantly elevated (Figure 2A). These data are consistent with the recent observation that the single *nrt1* mutant is more effective at cross feeding of the *qns1* strain than is a wild-type strain [11]. By comparison to *qns1* growth in SDC media supplemented with a known concentration of purified NR (Figure 2A), we estimate that the NR non-salvaging strain, PAB038, produces at least 1 µM extracellular NR when incubated to an OD of 3, whereas the NR non-salvaging and NR non-importing strain, PAB076, produces at least 2 µM extracellular NR under the same growth conditions.

**Figure 3:**
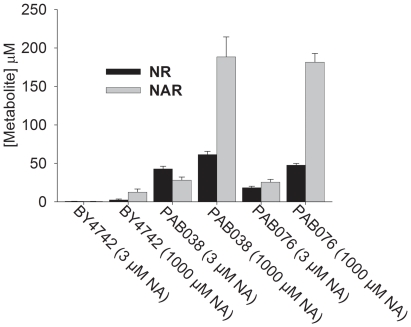
Intracellular NR is Reduced by *nrt1* Deletion and Intracellular NAR is Strikingly Increased by NA Supplementation. Intracellular NAD^+^ metabolite measurements by LC-MS indicate that *nrt1* deletion reduces intracellular NR and that NA supplementation particularly increases the intracellular NAR fraction.

**Figure 4:**
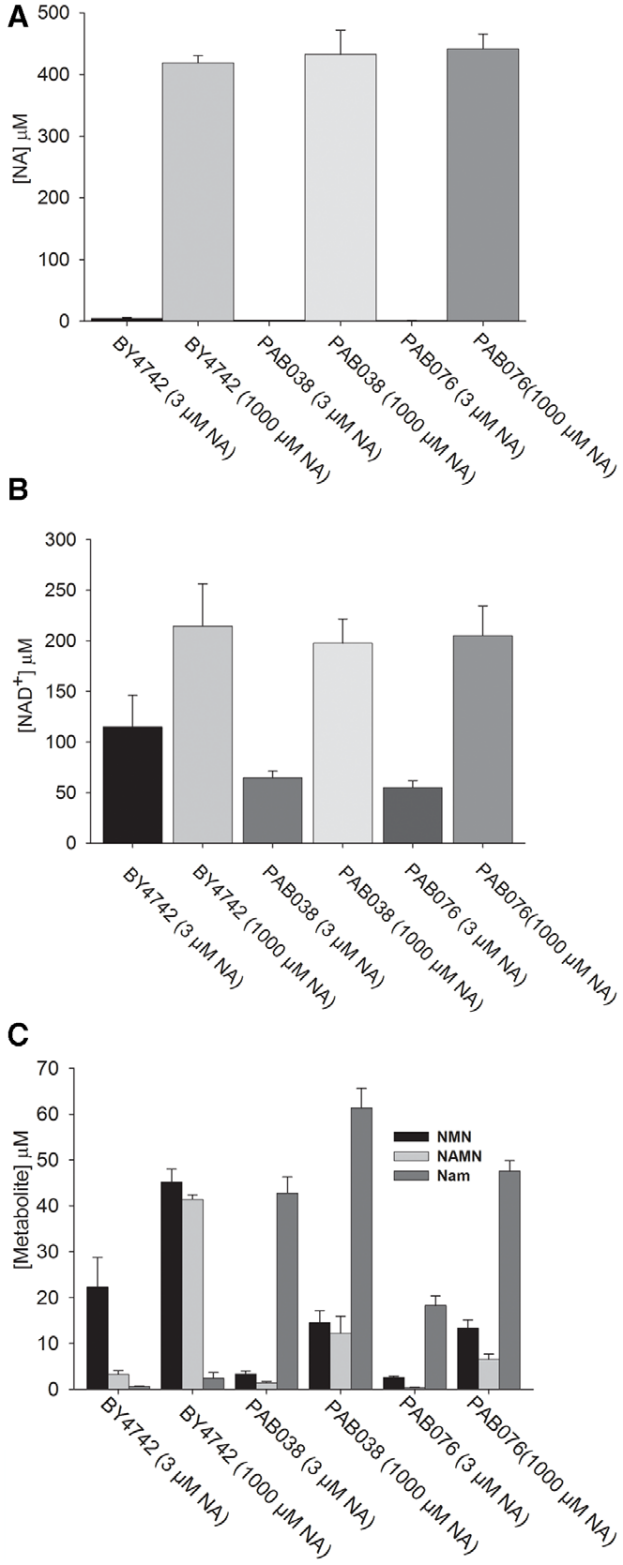
The intracellular NAD^+^ metabolome assayed by LC/MS. Intracellular NAD^+^ metabolite measurements by LC-MS in lysates of the indicated strains grown in SDC media supplemented with 3 µM or 1000 µM NA.

We hypothesized that excessive NR accumulates in the media of *nrt1* mutants because NR export is Nrt1-independent. If strain PAB076 exports NR that cannot be re-imported, this would result in higher extracellular and reduced intracellular NR in this strain. To test this hypothesis, we assayed the intracellular concentrations of the core NAD^+^ metabolome using a recently developed LC-MS assay [16], [17]. We found that the concentration of NR in yeast lysates is reduced by ∼57% from 42.7±3.5 µM to 18.2±2.0 µM when *NRT1* is deleted from the NR-nonsalvaging strain (Figure 3). All other NAD^+^ metabolites including NAR are unaffected by deletion of *NRT1* (Figure 3, Figure 4). These data are consistent with increased net export of NR in the transporter-free strain. Moreover, these data indicate that the NR transporter, Nrt1, functions in NAD^+^ metabolism even if cells are not specifically supplemented with NR.

### Supplementation with high concentrations of NA or Nam and growth to high cell densities increases NR export

NR has the potential to become a human dietary supplement and/or drug for prevention of neurodegenerative conditions or the treatment of dyslipidemia [14]. One hurdle to the development of NR as a product for human consumption has been the difficulty and expense of enzymatic or chemical synthesis [9], [12]. Improved NR export from yeast may provide a simple biological alternative to the current modes of NR production. Strain PAB076 is an excellent candidate for biological production of NR because it produces more NR than any strain identified to date. One possible method to increase NR export from PAB076 would be to supplement yeast with the inexpensive NAD^+^ precursors, NA or Nam. Supplementation with NA or Nam would replenish the NAD^+^ lost in production and export of NR. In addition NA supplementation leads to the overexpression of Isn1, the NR-producing 5′ nucleotidase [16], which may lead to higher NR production.

We used the *qns1* bioassay (Figure 2B) to survey the accumulation of NR in media conditioned by PAB076 in the presence of 1 mM NA or 1 mM Nam. Supplementation substantially increased the amount of NR produced. The extent of *qns1* growth was higher than growth provided by supplementation of SDC with 3 µM NR (Figure 2A–B), indicating that the concentration of NR in conditioned media was greater than 6 µM. To quantify extracellular NR, we employed MALDI-MS with a spiked-in ^18^O-labeled NR standard. We found that wild-type yeast exported NR to a level of 0.12±0.4 µM (Table 2). By comparison, the NR non-salvaging PAB038 strain increased NR accumulation ten-fold to 1.2 µM±0.4 µM and further deletion of *NRT1* in strain PAB076 increased NR accumulation another ∼three-fold to 4.0±0.9 µM (Table 2). Growing strains PAB038 and PAB076 in the presence of 1 mM NA increased NR accumulation to 3.9±1.5 µM and 7.7±1.1 µM, respectively. Growing the NR-exporting strain in 1 mM Nam or supplementing with both NA and Nam did not further improve NR production above the levels produced by PAB076 supplemented with 1 mM NA.

**Table 2:** Vitamin supplementation and growth to high OD increase NR production.

Strain	Media	Supplement	Optical Density	[NR] (µM)
BY4742	SDC	3 µM	3	0.12±.4
PAB038	SDC	3 µM	3	1.20±0.4
PAB038	SDC	1 mM NA	3	3.90±1.5
PAB076	SDC	3 µM	3	4.06±0.9
PAB076	SDC	1 mM NA	3	7.70±1.1
PAB076	SDC	1 mM Nam	3	7.17±0.2
PAB076	SDC	1 mM NA, 1 mM Nam	3	7.30±0.3
PAB076	YPD	1 mM NA	15	10.6±5.6
PAB076	2x YPD	1 mM NA	21	21.1±4.6
PAB076	SDC	5 mM NA	7	16.8±0.3
PAB076	2x SDC	5 mM NA	13	20.8±4.2
PAB076	2x YPD	5 mM NA	60	28.2±8.5

We also quantified intracellular NAD^+^ metabolite levels using LC-MS in strains incubated with 1 mM NA. We found that intracellular levels of NA, Nam, NAR, NaMN, NMN and NAD^+^ are elevated by supplementation with 1 mM NA in all strains tested (Figure 3, Figure 4). This is evidence for an increase in net NAD^+^ biosynthesis [17]. Interestingly, the increase in intracellular NR content of cells supplemented with 1 mM NA was substantially less than the increase of intracellular NAR and other elevated NAD^+^ metabolites, consistent with the exit of this compound from cells.

**Figure 5:**
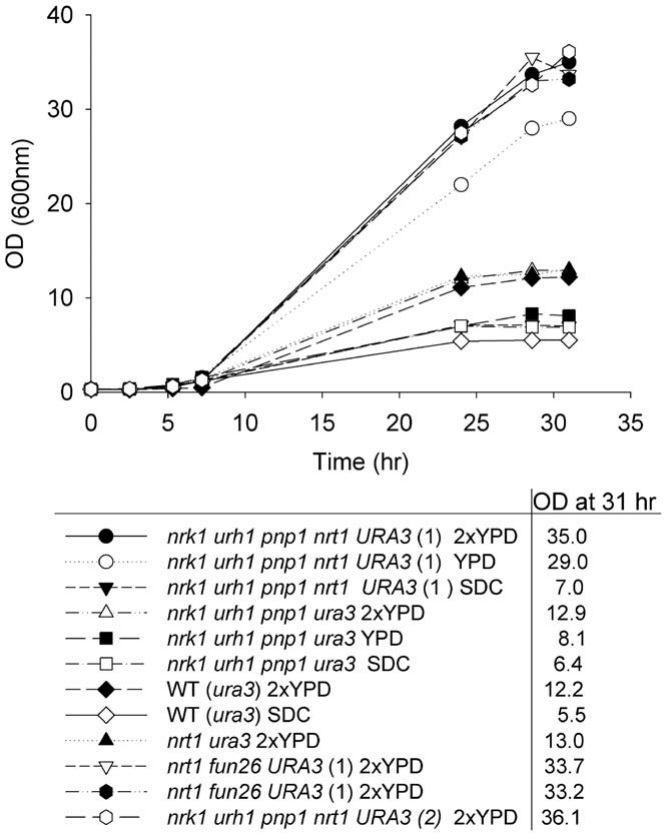
High density growth depends on *URA3*. The indicated strains were grown in the indicated media. Cell density was followed by OD_(600 nm)_. The data indicate that strains that are wild-type for *URA3* grow to higher density than *ura3* mutants.

By adding NA or Nam, we were able to double accumulation of extracellular NR. To further increase production of NR, we examined variables such as genotype, culture time, composition of media, and cell density. We incubated PAB076 in YPD, 2x YPD, SDC and 2x SDC media and measured cell density over time. Surprisingly we found that at 31 and 48 hours, PAB076 grew to an OD600 nm of 36 and 60 respectively. This cell density was 5 times higher than the maximum achieved by the other strains. To explain this phenotype, we incubated multiple related strains under the same conditions and tracked their growth for 31 hours (Figure 5). We found that high density growth was only observed in strains that are prototrophic for uracil. Thus, replacement of *NRT1* with *URA3* was fortuitous because *URA3* prototrophy allows the strain to grow to higher cell density.

Testing this strain, we found NR accumulation was related to increased cell density (Table 2). The highest NR accumulation was achieved in cells grown in 2x SDC with 5 mM NA and with 2x YPD supplemented with 5 mM NA. These cultures had NR levels of 20.8±4.2 and 28.2±8 µM at OD600 nm of 13 and 60, respectively (Table 2).

### Extracellular Nam is converted into extracellular NA in a Pnc1 dependent manner

**Figure 6:**
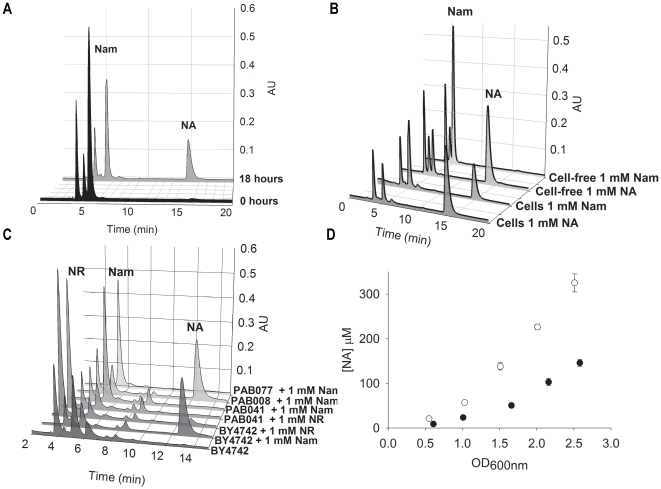
Extracellular Nam is Converted into Extracellular NA in a Pnc1-Dependent Manner. A) PAB076 cells grown in SDC media supplemented with 1 mM Nam produce a strong HPLC peak of extracellular NA after 18 hour of incubation. Two chromatograms are presented: one taken before exposure to PAB076 and the other after 18 hours of incubation. B) To determine whether Nam can be converted to NA simply by exposure to cell-free conditioned culture media, media supplemented with 1 mM NA or 1 mM Nam were analyzed by HPLC after exposure to cells or cell-free conditioned media. The data indicate that Nam is stable in cell-free conditioned media and depends on cells for conversion to NA. C) HPLC traces of media from yeast strains grown with Nam or NR supplementation. The absence of an NA peak in the PAB041 and PAB077 strains indicates that NA production is *PNC1* dependent. The robust NA peak in the PAB008 strain indicates that NA export is *TNA1*-independent. D) Quantification of extracellular NA content in media from WT cells (dark circles) and *tna1* cells (open circles) grown in SDC media supplemented with 1 mM Nam. The data indicate that cells without a transporter of NA accumulate greater extracellular NA.

Examination of the culture media from the NR-exporting strain, PAB076, when grown with 1 mM Nam suggested that the strain depletes Nam and accumulates extracellular NA after overnight incubation (Figure 6A). To test whether the Nam to NA conversion is cell-dependent or independent, we incubated 1 mM Nam or NA with the parental laboratory strain, BY4742, or in conditioned media collected from BY4742, for 18 hours. Under these conditions, NA levels were stable but Nam was converted to NA in the presence of cells (Figure 6B). However, Nam was not converted to NA by incubation with conditioned cell-free media collected from the same cells. This indicates that the Nam to NA conversion is cell-dependent and not the result of an extracellular enzyme activity. We therefore tested the hypothesis that Pnc1, the intracellular nicotinamidase [5], is required for production of extracellular NA from supplemented Nam.

As shown in Figure 6C, conversion of extracellular Nam to extracellular NA was completely abolished in strain PAB041 containing a *pnc1* mutation. This figure also shows that extracellular NR is converted to extracellular NA in a Pnc1-dependent manner. However, the final amount of NA produced form 1 mM extracellular NR is substantially less than the amount of NA produced from 1 mM Nam, presumably because a substantial fraction of NR metabolism flows to NMN production (Figure 1) [9].

Metabolism of extracellular Nam to extracellular NA requires two independent transport events: Nam import and NA export. To test whether the high affinity NA transporter, Tna1, is required for NA efflux, we examined extracellular Nam to NA conversion in the parental wild-type strain BY4742 versus a *tna1* mutant strain, PAB008. As shown in Figure 6C, PAB008 produced higher levels of NA than BY4742, indicating that Tna1 is not required for NA efflux. The fact that the transporter mutant strain exhibited greater NA accumulation indicates that PAB008 has higher net export of NA because of a reduction of high affinity NA import by the *tna1* deletion. A time course of NA accumulation in the two isogenic strains supports this mechanism (Figure 6D): deletion of Tna1 promotes increased net NA accumulation.

### Purification of NR from PAB076-conditioned media

**Figure 7:**
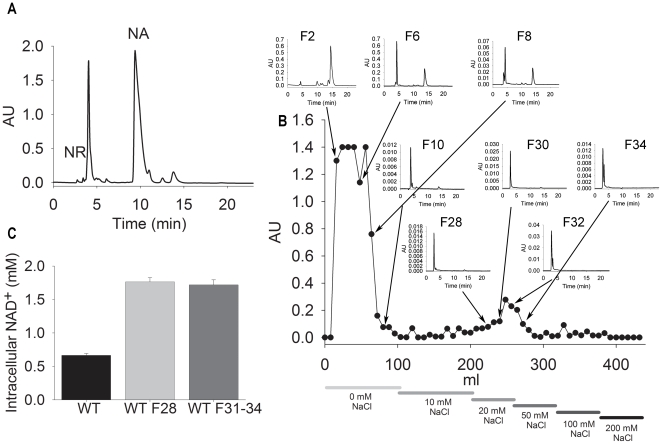
Purification of NR from PAB076-Conditioned Media. A) HPLC trace of media collected from strain PAB076 grown to OD 60 in 2x YPD and supplemented with 5 mM NA. B) Preparative SP-Sephadex chromatography with fractions analyzed by HPLC. NR eluted at 20 to 50 mM NaCl in fractions 27 to 36. C) Intracellular NAD^+^ determination of strain BY4742 grown in NA-free SDC media and NA-free SDC media supplemented with 10 µM NR from fractions produced in panel B.

To determine if the biology of bidirectional NR transport could be exploited to produce NR, we grew a 150 ml culture of PAB076 in 2x YPD, supplemented 5 mM NA, to an OD600 nm of 60 (Figure 7A). To extract NR from culture medium, we implemented a two-step process: concentration of NR by lyophilization/methanol extraction and separation of NR *via* SP-sephadex chromatography in a NaCl gradient (Figure 7B). NA and the majority of UV-absorbing media components eluted in the first 100 ml of fractions (0 mM NaCl). NR was retained by the resin and eluted between 20 and 50 mM NaCl in fractions 27 to 36. The majority of these fractions were >98% pure NR, although early fractions contained trace amounts of NA. Each fraction was concentrated by lyophilization and NR was quantified by absorbance at 259 nm [8]. Total recovery was ∼700 µg of NR (5.6 mg/l culture), which represented a 70% yield of the cultural concentration of NR (8 mg/l) as determined by MALDI-MS. Biological assays of NR from fraction 28 and from pooled fractions 31 to 34 were indistinguishable from chemically or enzymatically synthesized NR [9], [10] (Figure 7C). Process optimization steps, such as continuous fermentation and/or extraction, have not yet been examined. Further genetic improvements might include engineered overexpression of the NMN 5′-nucleotidase activities [16].

## Discussion

We set out to determine the role of the NR-specific transporter, Nrt1, in the efflux of NR by NR-nonsalvaging yeast cells and found that the transporter is not responsible for metabolite export. This initial experiment led us to identify three novel aspects of vitamin metabolism in yeast. First, neither the NR transporter, Nrt1, nor the NA transporter, Tna1, is responsible for efflux of the specific metabolite and deletion of each transporter leads to increased accumulation of the metabolite into culture media. Second, we discovered that yeast cells deficient in both NR salvage and NR import produce large quantities of extracellular NR. Based on this observation, we developed a novel method to produce and purify NR with the PAB076 strain, optimized by NA supplementation, growth and media conditions. Third, we found that extracellular Nam is rapidly converted to extracellular NA in a manner that depends on intracellular Pnc1 activity. These specific findings are each surprising and the extracellular nature of NAD^+^ metabolism in the unicellular fungus, *S. cerevisiae*, was not anticipated.

Whether a specific gene product is responsible for NR and NA export remains a mystery. Candidates for this activity include the group of multidrug resistance transporters of which there are more than 25 predicted members in yeast [18]. Multidrug resistance efflux pumps include the ATP-binding cassette superfamily and the MFS transporters [19]. One of the most striking characteristics of these exporters is the apparent lack of specificity for their transported cargo [19], [20], [21]. For example, yeast Pdr5 has as many as three active sites, each required for recognition of different groups of molecules [20]. In many cases, molecular size is the only specificity determinant [20]. It is therefore not unlikely that NR and NA efflux is accomplished by the nonspecific activities of multiple gene products.

The unanticipated export of NA and NR by yeast cells may play an important role in the regulation of intracellular NAD^+^ homeostasis. Export of vitamins may regulate intracellular enzyme activities by keeping substrate levels within an optimal range. For example, by converting Nam into NA and then exporting the product, cells may be deploying a mechanism to reduce the inhibitory effect of Nam on Sirtuins [5], [6]. In addition, transport to culture medium may be a reserve mechanism to store vitamins for later use and to feed other members of the colony. The permissivity of vitamin export without a specific transporter and he apparent transcriptional derepression of NRT1 and TNA1 [7], [10] at low sirtuin activity make possible a system in which excessive precursors are stored outside cells and taken up by other cells when needs arise [22]. Because cell to cell communication plays such important roles in metazoans, it will be interesting to look for nutrient-regulated vitamin export in other systems.

## Materials and Methods

### Yeast strains and media

All *S. cerevisiae* strains used in this study were derivatives of the laboratory strain, BY4742. Construction of single gene deletion strains has been described [23]. Additional deletions were created by direct transformation with PCR products [24]. Strain genotypes are in Table 1. Sequences of oligonucleotide primers are provided in supplementary materials.

NA-free synthetic dextrose complete (SDC) medium and its vitamin-supplemented forms have been described [9], [25]. 2x SDC and 2x yeast extract/peptone/dextrose (YPD) media were prepared as the more concentrated forms of the common preparations.

### NR bioassay

Strain BY165-1D, containing a deletion of the *qns1* gene and carrying the *URA3-*based *QNS1* plasmid pB175 [26], was plated on SDC with 0.1% 5-fluoroorotic acid and 10 µM NR to select for loss of pB175 [8]. The resulting strain was maintained on NR-containing media at all times. Conditioned media were prepared by incubating the specified yeast strains in the indicated media. After 18 hours, cells were removed by centrifugation and filtration. Conditioned media were retained and mixed in a 1∶1 ratio with fresh 2x SDC. Plasmid-free BY165-1d was incubated in the resulting media and NR-dependent growth of this strain was measured by optical density.

### Metabolite quantification

NR content in conditioned media was measured using matrix-assisted laser desorption ionization (MALDI) MS. A heavy standard of NR was synthesized from hydrolysis of cyanopyridine in ^18^O water to produce ^18^O Nam [27], followed by chemical synthesis of heavy NR from heavy Nam [28]. Prior to MALDI-MS measurement, ^18^O NR was added to media to a final concentration of 10 µM as an internal standard. 1 µl of conditioned media was then mixed with 1 µl of 50% acetonitrile saturated with 2,5-dihydroxy benzoic acid matrix and allowed to air-dry. Mass spectra were collected on an ABI Voyager-DE Pro MALDI-time of flight MS and the ratio of the labeled standard to unlabeled NR was used to determine the NR concentration. To quantify levels of the intracellular NAD^+^ metabolome, we utilized an LC-MS method as described [16], [17]. Extracellular NA, Nam and NR were also measured using HPLC. Media samples were injected directly onto a PrincetonSPHER60 SAX 5 µm (250×4.6 mm) column and separated isocratically with 20 mM KH2PO4 as the mobile phase. Metabolites were detected by absorbance at 260 nm and quantified by comparison to a standard curve.

### NR extraction and purification

PAB076 was cultured in 500 ml of 2x YPD to an OD_600 nm_ of 60 (∼60 hours). Media samples were frozen at −80°C in 150 ml portions and lyophilized. Each pellet was then resuspended in 25 ml of cold methanol, which solubilized the NR but left the majority of the contaminants in the pellet after centrifugation. Samples were then lyophilized again and resuspended in 5 ml of water. Aqueous samples were separated on a 10 ml SP-Sephadex column with NaCl as the mobile phase. Fractions were analyzed by HPLC. NR, which eluted at 25 to 50 mM NaCl, was confirmed by MALDI-MS, by support of growth of the *qns1* yeast strain [8], and, as shown in Figure 4D, by the ability to elevate intracellular NAD^+^ in wild-type yeast [9].

### Statistical methods

All measurements were performed at least three times. Data are reported as means of biological triplicates or quadruplicates ± standard deviations.
